# Lysophosphatidic Acid Level and the Incidence of Silent Brain Infarction in Patients with Nonvalvular Atrial Fibrillation

**DOI:** 10.3390/ijms11103988

**Published:** 2010-10-19

**Authors:** Zhen-Guang Li, Zhan-Cai Yu, Yong-Peng Yu, Wei-Ping Ju, Dao-Zhen Wang, Xia Zhan, Xi-Juan Wu, Li Zhou

**Affiliations:** Department of Neurology, Wendeng Center Hospital of Weihai, the Affiliated Hospital of Weifang Medical College, Weihai, Shandong 264400, China; E-Mails: lzg6598@163169.net (Z.-G.L.); wdchywk@163.com (Z.-C.Y.); jwpz67@126.com (W.-P.J.); zhenwd17@126.com (D.-Z.W.); wdzhx686@sina.com (X.Z.); wxj7979@163.com (X.-J.W.); drlizhou2009@126.com (L.Z.)

**Keywords:** atrial fibrillation, lysophospholipids, ischemic stroke, platelet activation

## Abstract

Lysophosphatidic acid (LPA), which is proposed to play an important role in normal physiological situations such as wound healing, vascular tone, vascular integrity and reproduction, may be involved in the etiology of some diseases such as atherosclerosis, cancer, obesity or myocardial infarction. Abnormal findings, including silent brain infarction (SBI), are frequently observed by magnetic resonance imaging (MRI) in patients with nonvalvular atrial fibrillation (NVAF). However, whether there is a relationship between LPA level and the prevalence of SBI has not been extensively studied. In the present study, the association between them was investigated. 235 patients with NVAF, 116 cases of SBI without NVAF and 120 cases of healthy volunteers (control group), who did not receive any antithrombotic therapy, were enrolled in this study. Plasma LPA levels in the NVAF with SBI group were significantly higher than that in the control group (*p* < 0.01), NVAF without SBI group (*p* < 0.01) and SBI without NVAF group (*p* < 0.01). The LPA levels are lower in the control group than in the NVAF without SBI and SBI without NVAF groups (*p* < 0.01), however, the latter two groups did not significantly differ from each other for LPA levels (*p* > 0.05) There were significant differences in the positive rate of platelet activation between each of the groups (*p* < 0.01). The positive rate of platelet activation was significantly higher in the NVAF with SBI group. We suggest that LPA might be a novel marker for estimation of the status of platelet activation and the risk factor for SBI onset in NVAF patients. We expected that plasma LPA levels could predict the occurrence of SBI in NVAF patients.

## 1. Introduction

Atrial fibrillation (AF) is the most common sustained arrhythmia and its incidence increases with age [1,2]. The incidence of stroke increased by 5–7 times in patients with AF in contrast to that with no AF [3,4]. Therefore, it seems particularly important to explore the mechanism of emboli formation in non-valvular atrial fibrillation (NVAF) patients. Previous studies mainly concentrated on the coagulation and fibrinolytic functions. However, few studies have addressed the platelet activation state in patients with NVAF *in vivo* since there are few molecular markers which can effectively reflect the platelet activation *in vivo* [5]. In recent years, studies have found that lysophosphatidic acid (LPA), as a multifunctional “phospholipid messenger”, is mainly produced by activated platelets and may be an ideal molecular marker [6,7] reflecting platelet activation state *in vivo*. Platelets contribute to the production of LPA [8], which has been proposed to be a primary lipid in atherosclerotic plaque that is responsible for platelet activation [9]. LPA is also abundant in human atherosclerotic plaques, where it is thought to be derived, at least in part, from mild oxidation of low density lipoproteins [10]. Platelet depletion or treatment with anti-platelet agents reduces circulating LPA levels in animals [11,12]. Hence, anti-thrombotic therapy targeting LPA production or receptor-mediated signaling may be a novel strategy to prevent thrombus formation. Silent brain infarction (SBI), which increases vascular cognitive impairment and stroke risk, has a high incidence in elderly people [13,14]. The occurrence of stroke in patients with NVAF is likely preceded by subclinical cerebrovascular disease. Many potentially abnormal findings, including the presence of SBI, which is detectable with brain magnetic resonance imaging (MRI), have been recognized in patients without clinically apparent previous cerebrovascular accidents [15–17]. Despite their high prevalence and clinical significance, the pathogenesis of silent ischemic lesions still remains elusive, especially in patients with NVAF [18]. There is consensus that it is high in elderly subjects and patients with risk factors for stroke such as hypertension, age and AF [16,19]. As with coronary artery disease, multiple observational or cross-sectional studies have shown a relationship between homocysteine (Hcy) and stroke [20–24]. In addition, a Japanese study found a relationship between Hcy and silent areas of infarction by MRI, that is to say, patients with a silent stroke also had high Hcy levels [14]. Other studies reported the incidence of SBI to be 2.5 to 4.5 times higher in hyperhomocysteinemia [14], which is an independent risk factor for vascular diseases such as cerebrovascular and cardiovascular diseases [25,26]. Furthermore, SBI has been considered as a risk factor for brain hemorrhage and symptomatic infarction [19]. Futoshi *et al.* [27] indicated that there was a significant association between plasma homocysteine level and the prevalence of SBI in hemodialysis patients with chronic renal failure. However, the significance of increased plasma LPA with SBI in NVAF patients has not been adequately investigated. We hypothesized that increased levels of LPA are associated with SBI in NVAF patients, which might provide basis for clinical antithrombotic therapy targeting LPA production and its clinical application.

## 2. Results and Discussion

### 2.1. Results

#### 2.1.1. Baseline Characteristics of the Study Groups

The baseline characteristics of each gGroup are shown in Table 1. Clinical and laboratory data, including age, sex, the occurrence of hypertension, diabetes mellitus and hyperlipidemia, and smoking status were not different in the four groups.

#### 2.1.2. Comparison of Plasma LPA Levels between Different Groups

**Figure 1:**
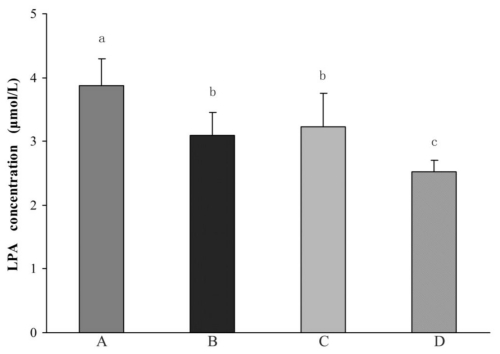
Comparison of LPA concentration (μmol/L) between the different study groups: (**A**) NVAF with SBI; (**B**) NVAF without SBI; (**C**) SBI without NVAF; (**D**) Control. Data are presented as means ± SEM. Columns relevant to each group bearing different superscripts (a, b or c) differ significantly (*p* < 0.01).

Among 235 cases of NVAF, 74 cases with SBI, which were found in accordance with the diagnostic criteria mentioned above, accounted for 31.5%. Plasma LPA levels in the NVAF with SBI group were significantly higher than that in the control group (*p* < 0.01), NVAF without SBI group (*p* < 0.01) and SBI without NVAF group (*p* < 0.01). The LPA levels are lower in the control group than in the NVAF without SBI and SBI without NVAF groups (*p* < 0.01). However the latter two groups were not significantly different from each other for LPA levels (*p* > 0.05) (Figure 1).

#### 2.1.3. Incidence of NVAF with SBI

There were 59 cases of NVAF with SBI out of 157 cases of NVAF in patients older than 60 years of age, which accounted for 37.6%. In contrast, there were 15 cases of NVAF with SBI in 78 cases of NVAF patients younger than 60 years of age, which accounted for 19.2%. For the control group, this percentage was 10.8%. However, adjusting for age, the difference in the incidences of SBI in patients with NVAF older than 60 years of age compared with control groups remained statistically significant (*p* < 0.01).

#### 2.1.4. Comparison in the Positive Rate of Platelet Activation

The percentage of platelet activation classified into mild, moderate and severe degree in the NVAF with SBI group was 6.8%, 8.1% and 85.1% respectively. However these percentages were 46.0%, 34.8% and 21.2%, respectively, in the NVAF without SBI group. In parallel, the data was 22.4%, 12.1% and 65.5% in the SBI without NVAF group and 79.2%, 12.5%, 8.3% in controls. There was a significant difference in the positive rate of platelet activation in each group (*p* < 0.01).

### 2.2. Discussion

The atrium loses its effective systolic function and the blood clots in the left atrium at AF. Due to endothelial dysfunction, platelet activation, coagulation and fibrinolytic activity being abnormal, the body is in a state of hypercoagulability or prethrombotic and the left atrium, particularly in the left atrial appendage, easily form mural thrombus. Once the thrombus is detached, it causes thromboembolic complications, especially ischemic stroke [14]. According to previous study, though platelet activation was not directly involved in thrombosis in AF, it could play an important role in blood hypercoagulability [28]. The data in the present study indicated that plasma LPA levels were significantly increased in NVAF patients with SBI. It revealed that platelet activation existed in these patients and that this activation might contribute to further LPA generation, which would activate more platelets to form emboli or emboli rain in turns. The emboli lead to SBI. Thus LPA can be used as a possible molecular marker for estimating thrombosis or prethrombotic status in patients with NVAF.

A Japanese community-based study reported that the incidence of SBI in the population of 66-year-old elderly was 24.8% [29], while the incidence of SBI in the population of 65-year-old elderly was 28% in a cardiovascular health study [30]. Our study found that the incidence of SBI in NVAF patients of 60-years-old was 37.6%, significantly higher than that in the age-matched control group (10.8%). However, the prevalence of SBI in patients with NVAF in our study is higher than that in the common population [29]. It should be noted that this prevalence is related to the particular study subjects rather than true prevalence, because the present study has a possible selection bias inherent in this type of hospital-based study. The prevalence of SBI will be affected by the sensitivity of the examination technique and the composition of the study samples with regard to age, sex, race and vascular risk factors. In additional, some other serum markers such as homocysteine (Hcy) or brain natriuretic peptide (BNP), known to correlate with an increased incidence of SBI, might be involved in the alteration of LPA levels. Hyperhomocysteinemia also enhances platelet adhesion to endothelial cells, promotes growth of vascular smooth muscles cells, and is associated with higher levels of prothrombotic factors such as β-thromboglobulin, tissue plasminogen activator and factor VII coagulant (FVIIc) [27]. Although this issue was discussed controversially, previous study indicated that elevated plasma concentrations of BNP were associated with the presence of AF [31] while the associations between these serum markers and LPA levels had not been fully understood. The additional further research on the role that the interaction between these serum markers such as LPA, Hcy and BNP played in the prevalence of the SBI was desirable. Furthermore, AF was one of the most important risk factors for SBI. So the frequency of SBI in the present study was relatively higher than that in previous reports mentioned above. Moreover, previous studies reported that an irregular heart rate blunted nitric oxide (NO) synthesis and increased p-selectin expression on platelets, ultimately increasing the risk for SBI [32,33]. Irregular shear stress upon the vascular endothelium in patients with AF also may cause sclerotic changes which contribute to the attenuation of NO synthesis [34,35]. This hypothesis is further supported by several reports that the prevalence of SBI lesions is associated with atherosclerosis related factors such as aging, diabetes mellitus and hypertension [36–39]. If the NO attenuation hypothesis is true, the higher prevalence of SBI in the NVAF patients may be explained better.

One of limitations of our study was that the study design was not randomized. Secondly, an extension of the actual data by the determination of some additional already established markers for platelet activation such as granulocyte-colony stimulating factor (G-CSF), interleukin-6 and thrombopoietin (TPO), *etc*., might strengthen the result of this study. It was a pity that these markers were not determined in this study. Thirdly, certain diseases may influence activated LPA release, including inflammatory processes, hypercholesterolemia or diabetes mellitus. In additional, LPA was measured after SBI onset in this study. Whether high LPA can be a risk factor for SBI needs a prospective study. Future studies, therefore, need to include not only prospective or longitudinal LPA comparative analyses but also investigations that will determine whether plasma LPA levels are influenced by other medical conditions or biomarkers which affecting the production, secretion and circulation of LPA. Longitudinal studies are also required to assess how well LPA levels correlate with SBI and thus might be used as a marker for monitoring treatment, progression and recurrence.

## 3. Materials and Methods

### 3.1. Study Subjects

Subjects enrolled in the study were informed and gave consent. The protocol of the study was approved by the local ethics committees. Patients were selected from 894 patients with NVAF who had been screened in outpatient clinics in four teaching hospitals. Of the 894 patients, 235 cases with NVAF and 116 cases of SBI without NVAF, who did not receive any antithrombotic therapy, were recruited as candidates for the present study. 120 cases of healthy volunteers older than 60-years of age (control group) were enrolled in this study. The controls were selected by the same experienced clinician and were subjected to neurological examination, and were neurologically normal adults in regard to sinus rhythm, recruited during routine health check-ups at the neurology outpatient clinic. All subjects enrolled in this study underwent echocardiography and ultrasonography of the carotid arteries. The candidate criterion used to select 235 cases with NVAF is as following: (1) Continuous or intermittent NVAF in patients was diagnosed by clinical symptoms, electrocardiogram, cardiac ultrasound and so on; (2) Age varied from 45 to 85. Exclusion criteria: Patients with NVAF, who had any of the following illnesses, conditions or requirement: (1) Rheumatic or congenital valvular heart disease; (2) After heart valve replacement; (3) Congestive heart failure and severe heart diseases. The diagnosis of congestive heart failure and severe heart diseases was confirmed by a complete medical evaluation, medical history and physical examination as well as various tests including echocardiogram and a chest X-ray to detect abnormal function of the left ventricle and/or heart valves and the size and shape of the heart; (4) Coagulation abnormalities. Coagulation abnormalities was defined as an INR spontaneously >1.6 and/or an APTT >60 s and/or a platelet count <150 × 10^9^/L and/or a fibrinogen less than 1.0 g/L; (5) Intracranial or systemic infection; (6) Obvious organs dysfunction (such as liver, kidney, lung). The cut off values about organs dysfunction were shown in Table 2; (7) With previous history of stroke or transient ischemic attack (TIA); (8) Receiving antithrombotic or warfarin therapy; (9) Hypertension with carotid atherosclerosis or hemal stricture revealed by carotid color ultrasonic examination; (10) Female in the menstruation period. 116 cases of SBI without NVAF were selected from patients who were hospitalized with dizziness, headache, cervical spondylosis, lumbar spinal stenosis, intracranial infection, acute carbon monoxide poisoning, facial paralysis and so on. A normal neurological examination with no focal deficit at the time of entry was required. 120 healthy controls that had no history of cerebral or cardiac vascular diseases and no symptoms mentioned above within one month were matched to patients by age and gender.

Information about clinical characteristics in each group such as arterial hypertension (AH), diabetes mellitus (DM), dyslipidemia and smoking habit were recorded. Cranial MRI was performed in all participants at entry to the study. Because this study involved multiple institutions, the MRI instruments differed, and a magnetic field strength of 1.0 T or more was used at each institution. Almost all patients underwent MRI examination, except for two patients in the SBI group who had metal in their bodies, and were analyzed on the basis of cerebral computed tomography without MRI. All subjects were examined by the same investigator who was blinded to clinical characteristics. The diagnosis of SBI was made with reference to the criteria established by Matsui *et al.* [14,40]: (1) SBI was defined as an area (≥3 mm in diameter) of focal hyperintensity on T2-weighted images or FLAIR images with corresponding low signal intensity on T1-weighted images and low density lesions on CT; (2) Lack of evidences for the nervous system symptoms or signs which can be explained by lesions observed on MRI or CT; (3) Without a history of clinical stroke; (4) Patients with micro-bleeding or suspected bleeding followed by infarction were excluded by imaging.

### 3.2. Sample Collection and LPA Analysis

Patients were told to stay away from fatty food and alcohol for several days before fasting blood samples were obtained in the morning. We did not have blood drawn from women if they were in their menstruation period. Four milliliters of venous blood was drawn from each participant into commercially available anticoagulant tubes (Two-fish Comp.). Plasma was either processed immediately or stored at −70 °C before lipid extraction. Lipid extraction was performed at 0 °C to 4 °C to minimize damage to ester bonds, using a slight modification of published methods [41]. Whole blood was centrifuged at 3500 g for 10 minutes. The supernatant was transferred to a microcentrifuge tube and centrifuged at 8000 g for 10 minutes. Then 1 mL suspension (platelet-poor plasma) was used for the remaining procedure. Lipids were extracted with 4 mL of 1-butanol and 2 mL of water-saturated 1-butanol. The resulting organic extracts were pooled and dried *in vacuo.* Each sample was resuspended in 0.3 mL of chloroform/methanol/water/28% NH_4_OH (250:100:15:0.3, v/v) and was immediately filtered by an Econosphere 3 μm, 50 × 4.6 mm silica column (Alltech Associates, Deerfield, IL). Compounds were eluted with a mobile phase of chloroform/methanol/water/28% NH4OH (250:100:15:0.3, v/v) at 0.5 mL/min. The source was maintained at 250 °C with a drying gas flow of 10 L/h. The concentration of LPA was quantified by measuring its inorganic phosphorus component using colorimetric assays.

### 3.3. Estimation of Platelet Activation Degree

Plasma LPA was considered to be a more sensitive and meaningful biomarker of platelet activation and LPA level could indirectly reflect the degree of platelet activation *in vivo*. The cut-off values for LPA levels were classified according to the degree of platelet activation in this study as follows: If the LPA concentration ≤3.0 μmol/L, platelet function was considered as being in a state of normal or mild activation; If LPA concentration >3.0 μmol/L, but ≤3.5 μmol/L, the platelet function was in moderate activation state; LPA concentration >3.5 μmol/L, the platelet function was in a highly activated state or the formation of emboli. The data about the risk classification in respect to the LPA plasma levels is based on our previous study [42]. However, these cut-off values may be applicable only to this study population and will have to be reevaluated following larger studies.

### 3.4. Statistical Analysis

Comparisons of the baseline characteristics and observations in the present study were performed by ANOVA and univariate analysis with Student.s t-test or Mann-Whitney.s test for continuous variables and the Fisher exact test for noncontinuous variables. Chi-square test was used to analyze the incidence of SBI between the NVAF group and the control group. All statistical analyses were performed with SPSS software package for Windows version 11.5. Data was expressed as mean ± SEM and statistical significance was set at *p* < 0.05.

## 4. Conclusions

Our data indicate that the plasma LPA levels significantly increased in NVAF or NVAF patients with SBI, which was accompanied by the high incidence of SBI. Based on the results presented here, we suggest that LPA might be a novel marker for estimation of the status of platelet activation and the risk factor for SBI onset in NVAF patients. We expect that plasma LPA levels could predict the occurrence of SBI in NVAF patients. There may be a relationship between plasma LPA levels and the prevalence of SBI in NVAF patients. If the plasma LPA levels were not an independent predictor for SBI, its predicted significance may be limited and this data should be interpreted carefully because the present study was not designed to test the predictabilities of plasma LPA levels. However, our results warrant further study. This was also the first study to examine the association between plasma LPA levels and the occurrence of SBI in NVAF patients and it might be an important subject for randomized trials and prospective research for some years to come.

## Figures and Tables

**Table 1:** Baseline characteristics of each group.

Variable	NVAF + SBI (+) (n = 74)	NVAF + SBI (−) (n = 161)	SBI + NVAF (−) (n = 116)	Control (n = 120)	*p*
Age, years (mean + SD)	64.6 ± 6.5	65.6 ± 7.9	62.5 ± 7.6	66.9 ± 5.8	ns
Male	39 (52.7%)	83 (51.6%)	65 (56.0%)	64 (53.3%)	ns
Hypertension	38 (51.4%)	78 (48.4%)	59 (50.9%)	51 (42.5%)	ns
Diabetes mellitus	17 (23.0%)	36 (22.4%)	25 (21.6%)	22 (18.3%)	ns
Dyslipidemia	20 (27.0%)	40 (24.8%)	28 (24.1%)	26 (21.7%)	ns
Smoking	13 (17.6%)	27 (16.7%)	20 (17.2%)	18 (15.0%)	ns
Platelet count (×10^9^)	197.1 ± 38.7	180.3 ± 33.6	203.2 ± 40.5	190.4 ± 38.8	ns

NVAF with SBI: NVAF + SBI (+); NVAF without SBI: NVAF + SBI (−); SBI without NVAF: SBI + NVAF (−).

**Table 2:** The cut-off values for organ dysfunction.

Variables	Cut off Values
Respiratory, PaO_2_/FiO_2_ (mmHg)	≤300
Coagulation, platelets ×10^3^/μmol	≤150
Liver, bilirubin (mg/dL)^a^	≥2.0
Cardiovascular, hypotension	Mean arterial pressure <70 mmHg
Renal creatinine (mg/dL)^b^ or	>2.0 or
Urine output (mL/d)	<500 mL
Glomerular filtration rate (GFR)	≤70 mL/min

a To convert bilirubin from mg/dL to μmol/L, multiply by 17.1;

b To convert creatinine from mg/dL to μmol/L, multiply by 88.4.
